# Supplementary material for: The polyadenylase PAPI is required for virulence plasmid maintenance in pathogenic bacteria
Source: bioRxiv. 2024 Nov 8:2024.10.11.617751. Originally published 2024 Oct 11. Preprint. [Version 2] doi: 10.1101/2024.10.11.617751 (PMC11482874; doi:10.1101/2024.10.11.617751)
Supplement: Supplement 1 — Figure S1. A transposon insertion into pil leads to a constitutively high pYV PCN, elevated T3SS expression, and a severe growth defect under T3SS-inducing conditions. (A) HEK293T cells expressing an NFκB luciferase reporter were left uninfected (uninf) or were infected with an effectorless Δyop6 strain (ΔyopHEMOJ), a ΔyscNU T3SS-deficient strain, or pil::Tn in the Y. pseudotuberculosis IP2666pIB1 background. Averages of three independent replicates ± standard error the mean are shown (Student t-test, p<0.04). (B) Schematic representation of the location of pil. The pil locus is located ~123bp away from the copB gene. (C) Wildtype, pil::Tn, and Δpil IP2666pIB1 were grown at 37°C/low calcium and secreted T3SS cargo proteins (Yops) were precipitated and visualized by Coomassie blue staining. Bovine serum albumin (BSA) serves as a loading and precipitation control. Data shown is representative of three independent replicates. (D-E) Relative pYV PCN was estimated for strains in the YPIII/pIBX background of Y. pseudotuberculosis using a luciferase plasmid copy number assay. For each timepoint, luminescence was measured and normalized to cell density (OD600). Representative data from three independent experiments are shown. (E) Serial dilutions for wildtype YPIII/pIBX and the pil::pNQ congenic Y. pseudotuberculosis strains were spotted onto low calcium agar plates for ~16 hours at 37°C before imaging. The yellow arrow indicates an example of a large colony in the pil::pNQ mutant background that represents a candidate suppressor mutant. Figure S2. T3SS gene mRNA levels are higher in the pil::Tn mutant and lower in the ΔpcnB mutant compared to wildtype. Heat maps displaying relative expression of pYV-encoded genes from RNA-seq analysis of Y. pseudotuberculosis IP2666pIB1 strains grown at 26°C or 37°C/low calcium. Figure S3. The pil::Tn strain exhibits normal temperature and calcium regulation of T3SS activity. Strains were grown in regular LB or low calcium LB media, at eit [file media-1.pdf]

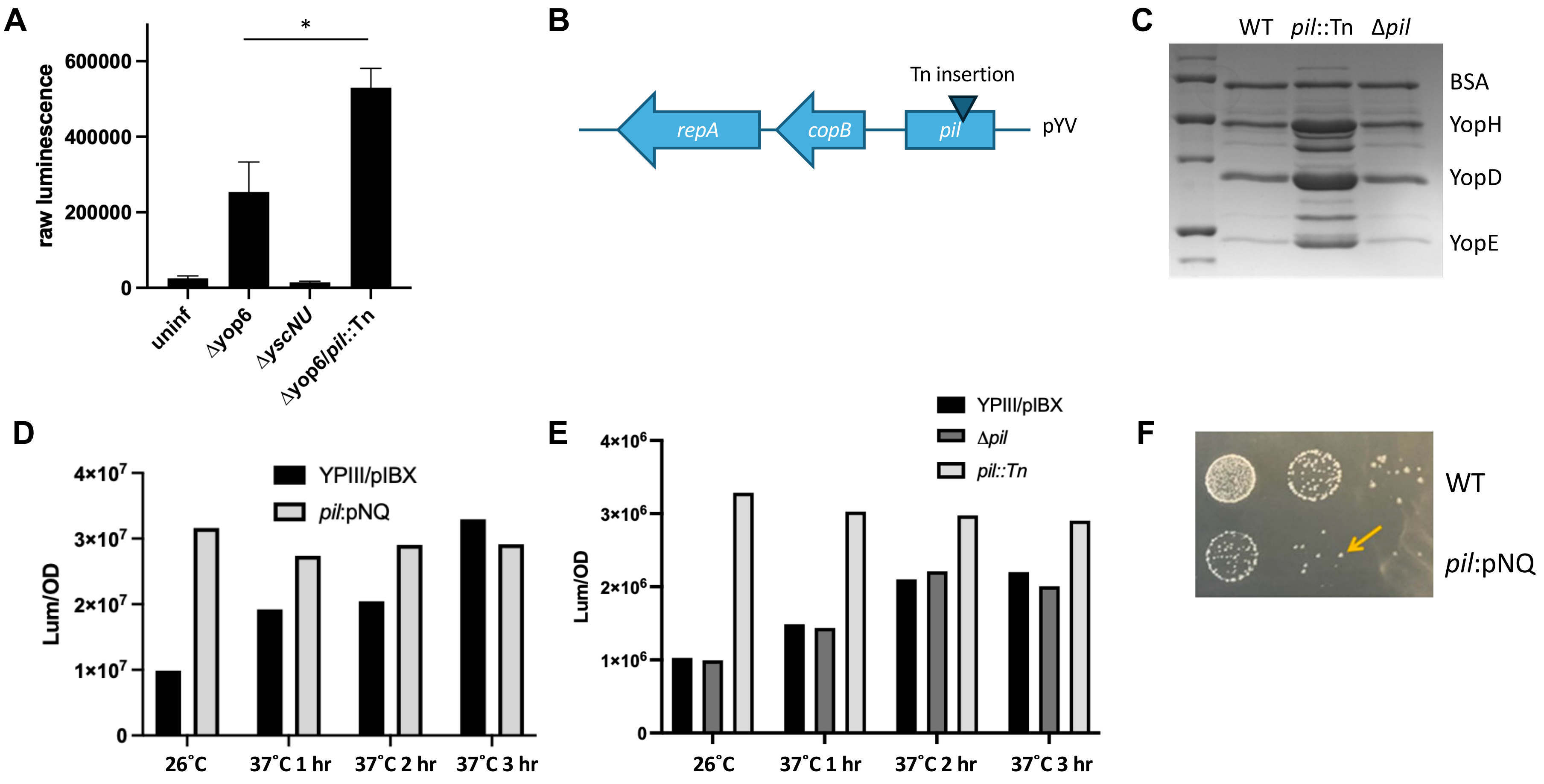
A
raw luminescence
600000
400000
200000
0
*
uninf
Δyop6
ΔyscNU
Δyop6/pil::Tn
B
Tn insertion
repA
copB
pil
pYV
C
WT
pil::Tn
Δpil
BSA
YopH
YopD
YopE
D
Lum/OD
4×10^7
3×10^7
2×10^7
1×10^7
0
YPIII/pIBX
pil:pNQ
26˚C
37˚C 1 hr
37˚C 2 hr
37˚C 3 hr
E
Lum/OD
4×10^6
3×10^6
2×10^6
1×10^6
0
YPIII/pIBX
Δpil
pil::Tn
26˚C
37˚C 1 hr
37˚C 2 hr
37˚C 3 hr
F
WT
pil:pNQ

Figure S1

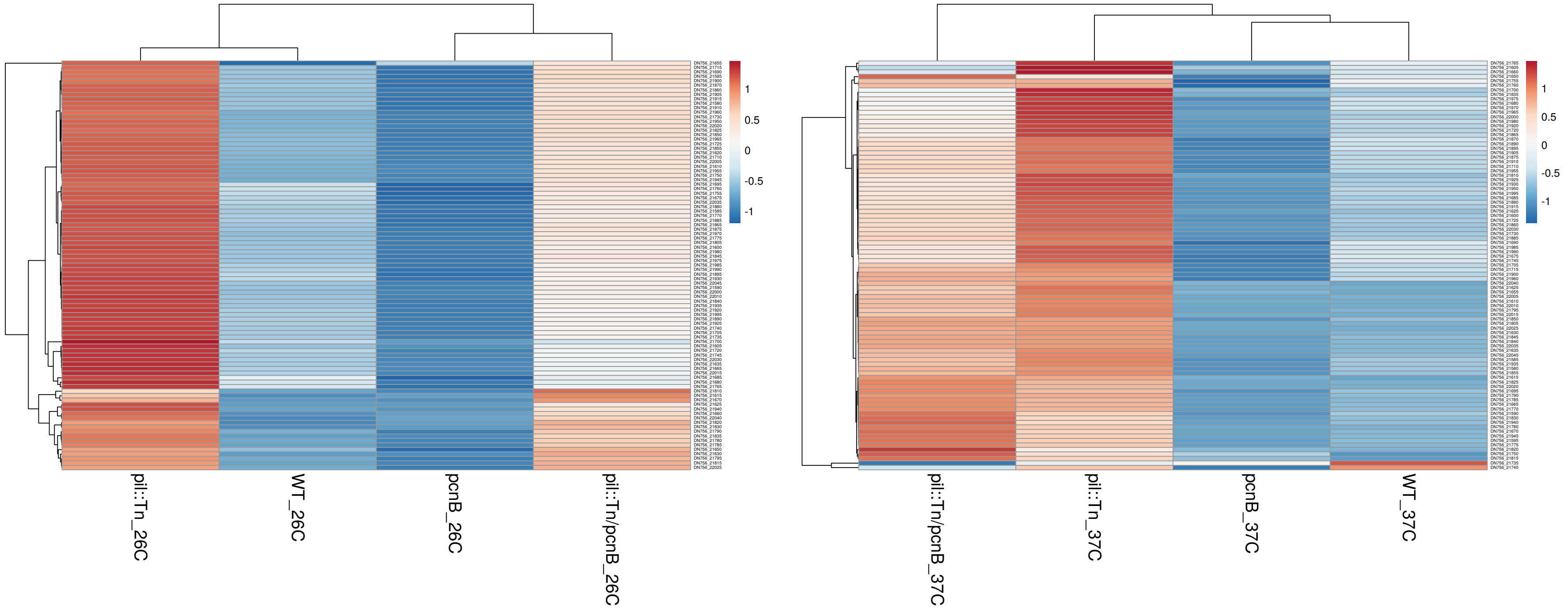
1
0.5
0
-0.5
-1
pil::Tn_26C
WT_26C
pcnB_26C
pil::Tn/pcnB_26C
1
0.5
0
-0.5
-1
pil::Tn/pcnB_37C
pil::Tn_37C
pcnB_37C
WT_37C

Figure S2

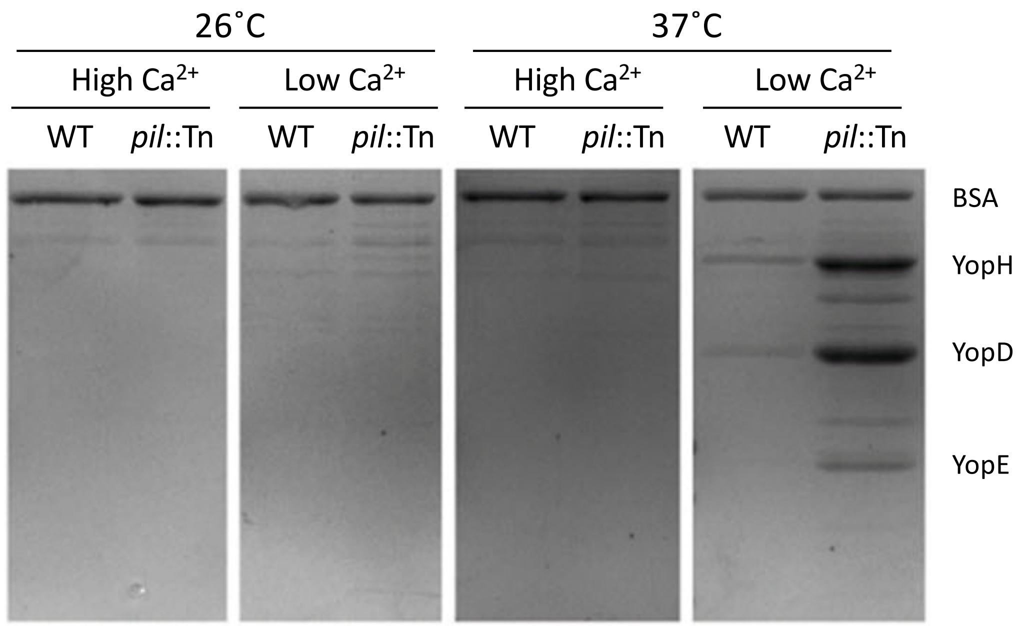
26˚C
37˚C
High Ca2+
Low Ca2+
High Ca2+
Low Ca2+
WT
pil::Tn
WT
pil::Tn
WT
pil::Tn
WT
pil::Tn
BSA
YopH
YopD
YopE

Figure S3

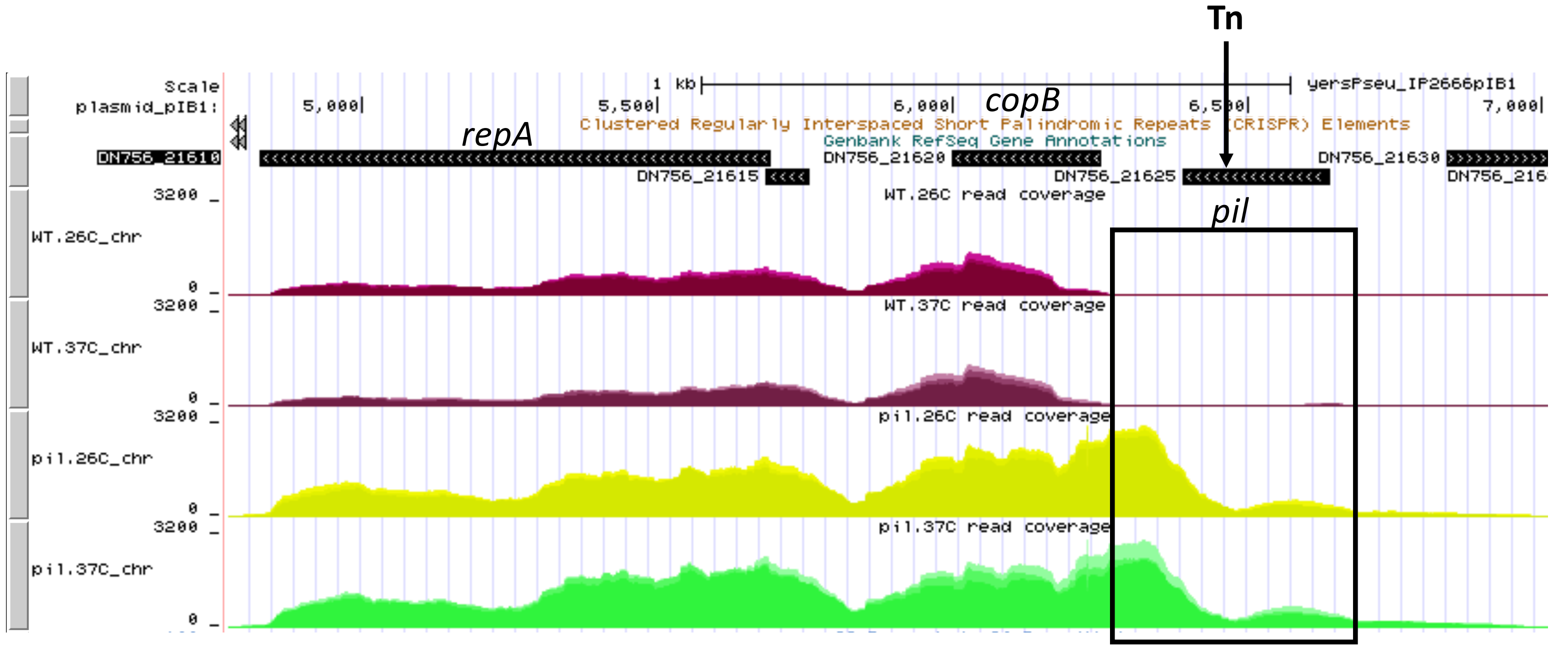
Tn
Scale
plasmid_pIB1:
1 kb
yersPseu_IP2666pIB1
5,000
5,500
6,000
6,500
7,000
copB
repA
Clustered Regularly Interspaced Short Palindromic Repeats (CRISPR) Elements
Genbank RefSeq Gene Annotations
DN756_21610
DN756_21620
DN756_21630
DN756_21615
DN756_21625
DN756_216
pil
3200
0
WT.26C read coverage
WT.26C_chr
WT.37C read coverage
WT.37C_chr
pil.26C read coverage
pil.26C_chr
pil.37C read coverage
pil.37C_chr

Figure S4

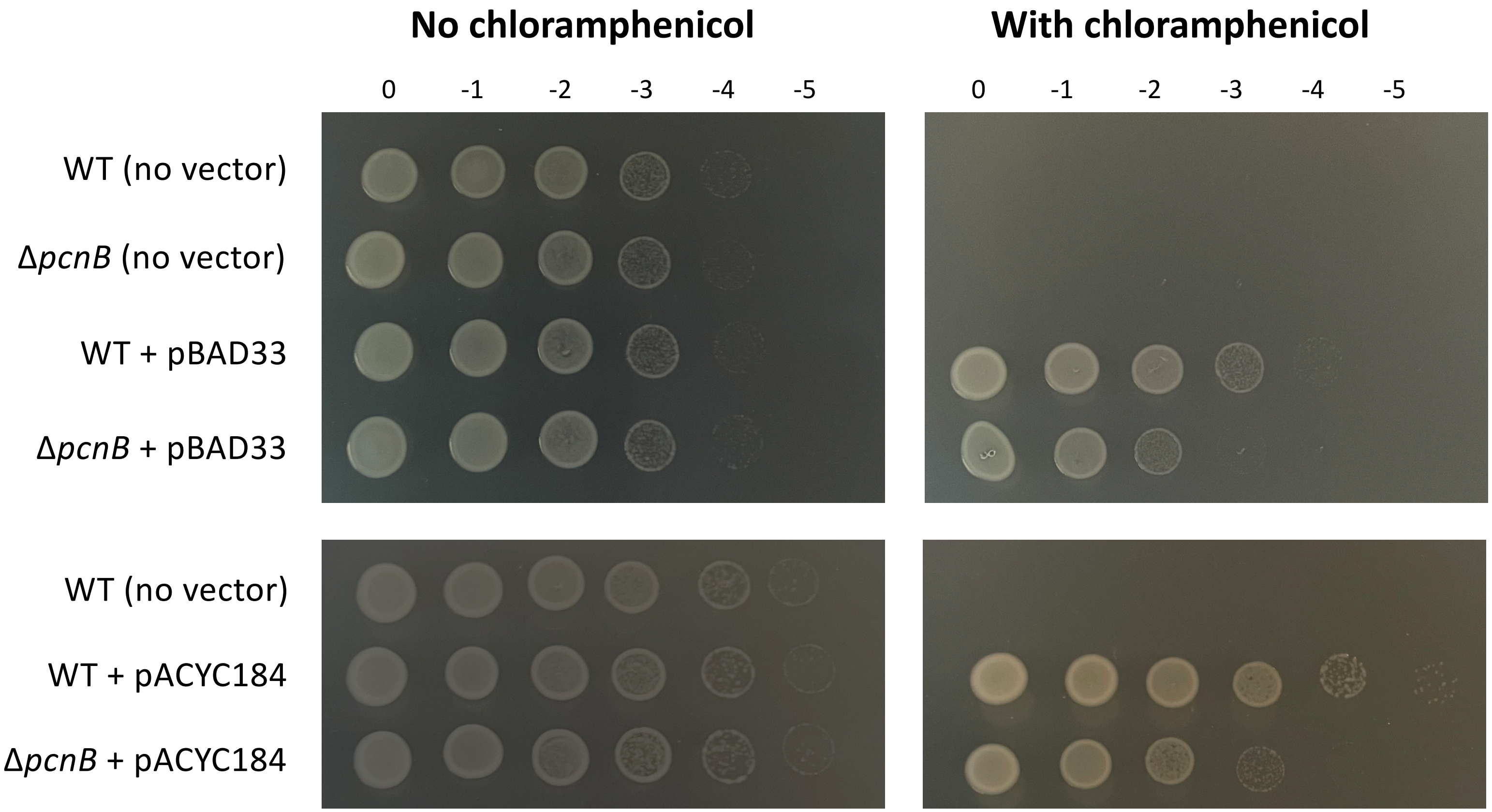

No chloramphenicol
With chloramphenicol
0
-1
-2
-3
-4
-5
WT (no vector)
ΔpcnB (no vector)
WT + pBAD33
ΔpcnB + pBAD33
WT (no vector)
WT + pACYC184
ΔpcnB + pACYC184

Figure S5

A

	**26˚C**	**37˚C**
WT	100%	94%
Δ*pcnB*	89%	39%
WT-3	100%	94%
WT-5	100%	67%
Δ*pcnB*-2-9	85%	16%
Δ*pcnB*-2-10	92%	25%
Δ*pcnB*-3-1	83%	8%
Δ*pcnB*-3-2	91%	23%

B

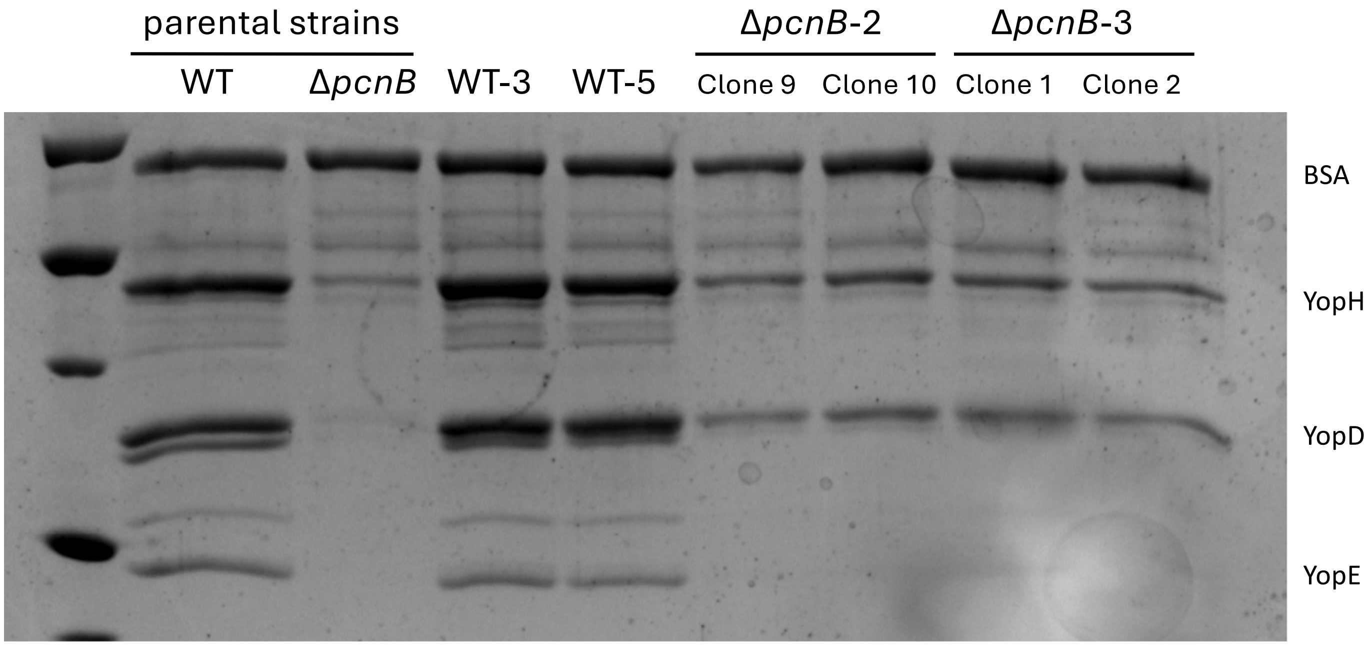

Figure S6

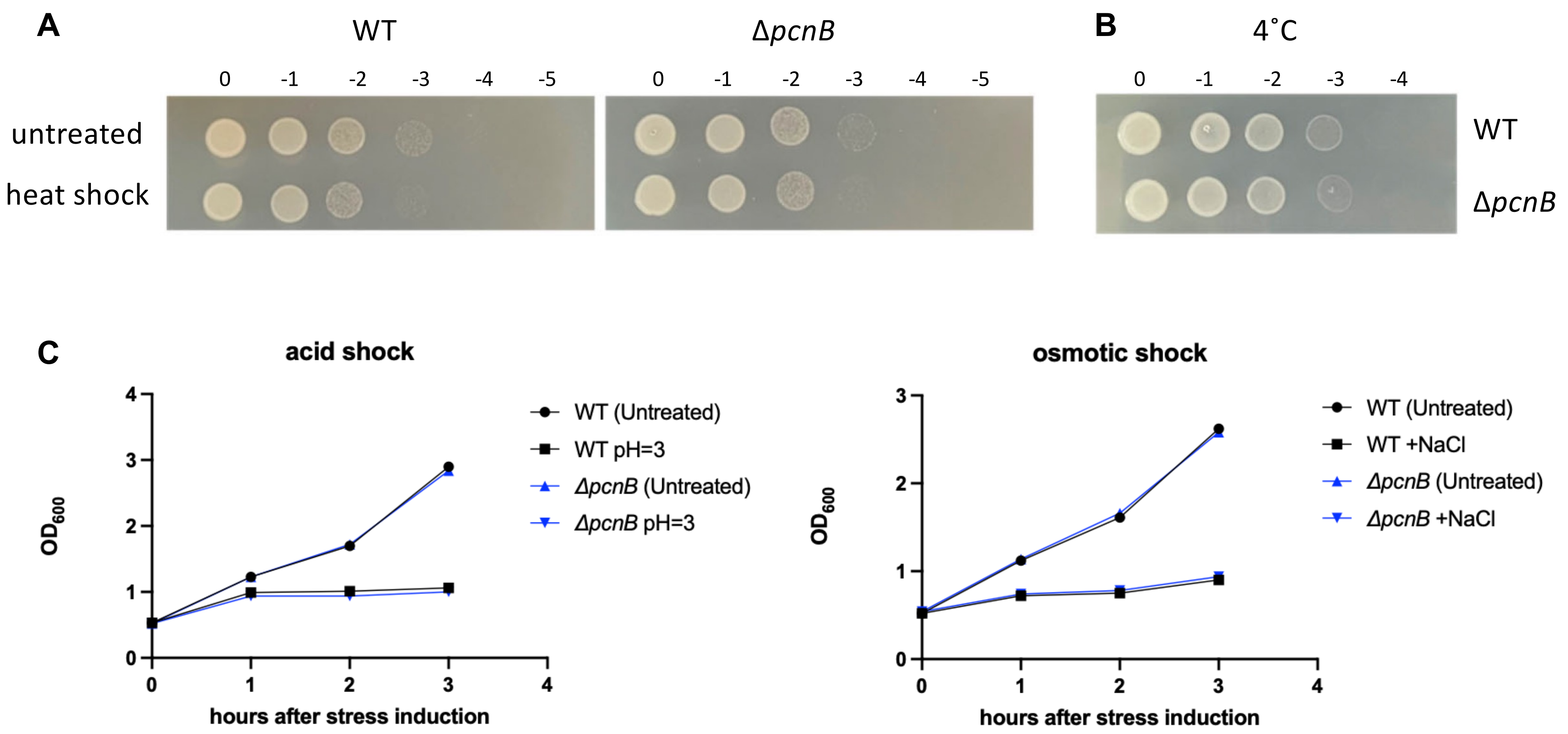
A
WT
ΔpcnB
0 -1 -2 -3 -4 -5
untreated
heat shock
B
4˚C
0 -1 -2 -3 -4
WT
ΔpcnB
C
acid shock
OD600
hours after stress induction
WT (Untreated)
WT pH=3
ΔpcnB (Untreated)
ΔpcnB pH=3
osmotic shock
WT (Untreated)
WT +NaCl
ΔpcnB (Untreated)
ΔpcnB +NaCl

Figure S7

```
Y.pseudotuberculosis(IP2666)      MFTRVANFCRKVLIREDKTVRDDKARKDKVPGEDNVARKERRPARAHTGRKGHAVSSSEQ  60
Y.pestis(Kim5)                    MFTRVANFCRKVLIREDKTVRDDKARKDKVPGEDNVARKERRPARAHTGRKGHAVSSSEQ  60
                                  ************************************************************

Y.pseudotuberculosis(IP2666)      RQMAIIPRDQHNISRRDISDNALKVLYRLNKSGYEAYLVGGGVRDLLLGRKPKDFDITTS  120
Y.pestis(Kim5)                    RQMAIIPRDQHNISRRDISDNALKVLYRLNKSGYEAYLVGGGVRDLLLGRKPKDFDITTS  120
                                  ************************************************************

Y.pseudotuberculosis(IP2666)      ATPEQVRKLFRNCRLVGRRFRLAHVMFGPEIIEVATFRGHHEQQQAEDSDKNSSQQAQNG  180
Y.pestis(Kim5)                    ATPEQVRKLFRNCRLVGRRFRLAHVMFGPEIIEVATFRGHHEQQQAEDSDKNSSQQAQNG  180
                                  ************************************************************

Y.pseudotuberculosis(IP2666)      MLLRDNIFGSIEDDAQRRDFTINSLYYGISDFALRDYTGGLRDLKEGIIRLIGDPETRYR  240
Y.pestis(Kim5)                    MLLRDNIFGSIEDDAQRRDFTINSLYYGISDFALRDYTGGLRDLKEGIIRLIGDPETRYR  240
                                  ************************************************************

Y.pseudotuberculosis(IP2666)      EDPVRMLRAVRFAAKLDMSISPETAEPIPRLASLLREIPPARLFEESLKLLQSGYGYKTY  300
Y.pestis(Kim5)                    EDPVRMLRAVRFAAKLDMSISPETAEPIPRLASLLREIPPARLFEESLKLLQSGYGYKTY  300
                                  ************************************************************

Y.pseudotuberculosis(IP2666)      LKLCEYQLFQPLFPLIARNFTEQHDSPMERILVQVLKNTDHRLHNDQRVNPAFLFAAMLW  360
Y.pestis(Kim5)                    LKLCEYQLFQPLFPLIARNFTEQHDSPMERILVQVLKNTDHRLHNDQRVNPAFLFAAMLW  360
                                  ************************************************************

Y.pseudotuberculosis(IP2666)      YPLIEHAQKLTQESGLAYYDAFALAMNDVLDEECRSLAIPKRITSLVRDIWLLQLRLSRR  420
Y.pestis(Kim5)                    YPLIEHAQKLTQESGLAYYDAFALAMNDVLDEECRSLAIPKRITSLVRDIWLLQLRLSRR  420
                                  ************************************************************

Y.pseudotuberculosis(IP2666)      QGKRAHKLMEHPKFRAAYDLLLLRAEVEKNHELQRLAQWWGEFQEATPTQQKSMLNTLGA  480
Y.pestis(Kim5)                    QGKRAHKLMEHPKFRAAYDLLLLRAEVEKNHELQRLAQWWGEFQEATPTQQKSMLNTLGA  480
                                  ************************************************************

Y.pseudotuberculosis(IP2666)      DPAPRRSRPRRPRKPVPRKEGV        502
Y.pestis(Kim5)                    DPAPRRSRPRRPRKPVPRKEGV        502
                                  **********************
```

Figure S8

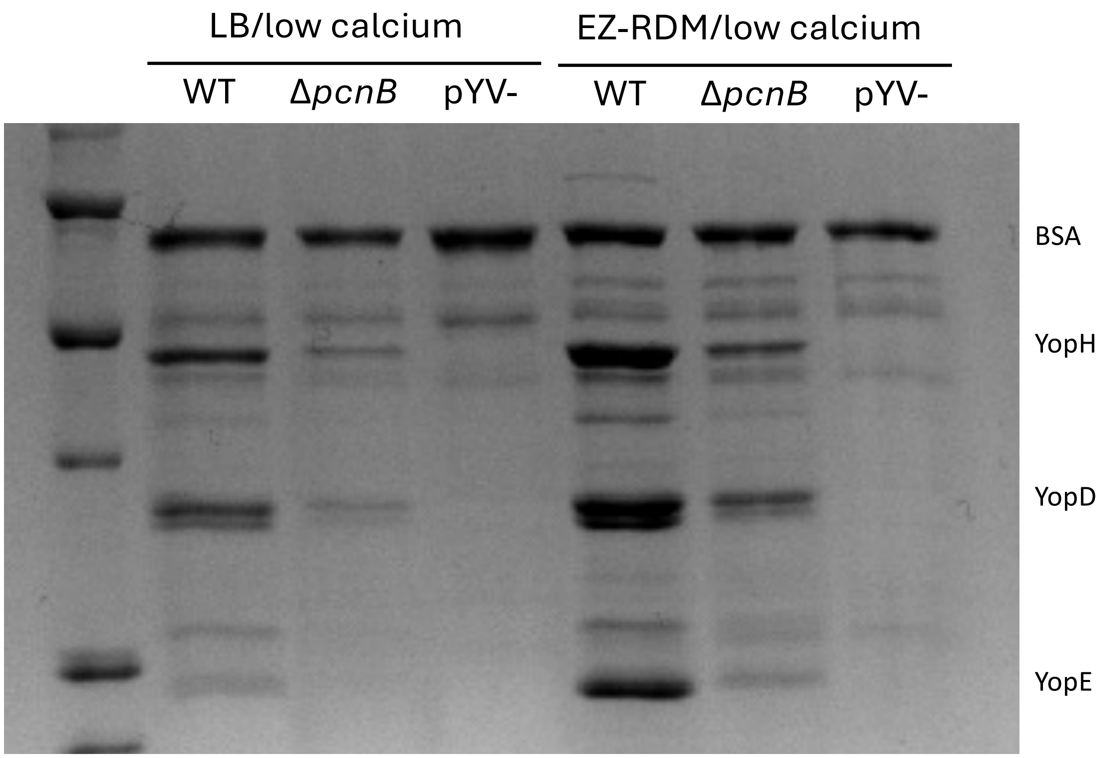

LB/low calcium
EZ-RDM/low calcium
WT
ΔpcnB
pYV-
WT
ΔpcnB
pYV-
BSA
YopH
YopD
YopE

Figure S9
