# Supplementary material for: The polyadenylase PAPI is required for virulence plasmid maintenance in pathogenic bacteria
Source: bioRxiv. 2024 Nov 8:2024.10.11.617751. Originally published 2024 Oct 11. Preprint. [Version 2] doi: 10.1101/2024.10.11.617751 (PMC11482874; doi:10.1101/2024.10.11.617751)
Supplement: Supplement 2 — Table S1. Phenotypic categories and mutations identified in suppressor screen. Table S2. Y. pseudotuberculosis strains used in this study. Table S3. Primers used in this study. Table S4. Plasmids used in this study. [file media-2.docx]

**Table S1. Phenotypic categories and mutations identified in suppressor screen**

| Strain: | PCN 26°C^1^ | PCN 37°C^1^ | T3SS activity^3^ | Identified Mutation: | Phenotypes: |
| --- | --- | --- | --- | --- | --- |
| pil::pNQ  (parental) | High^2^ | High ^2^ | High ^2^ | N/A |  |
| IS0002 | No Change | No change | No Activity | TGA 🡪 GGA mutation abolishing YscI stop codon | Predicted to decrease or abolish translation of YscI |
| IS0003 | No Change | Lower | Lower | G 🡪 C mutation in tRNA-Gly | Unknown how this mutation impacts pYV PCN and/or T3SS activity. |
| IS0004 | No Change | Lower | Lower | G 🡪 A mutation in *prepA* -35 region | Predicted to decrease  p*repA* promoter firing |
| IS0006 | Lower | Lower | Lower | L291R mutation in PAP I | Mutation lowers PAP I protein levels at 37°C |
| IS0007 | No Change | No Change | No Activity | K175E mutation abolishing the essential lysine in walker box A of YscN | Mutation predicted to impair YscN ATP binding and hydrolysis |
| IS0008 | Lower | Lower | Lower | L291R mutation in PAP I | Mutation lowers PAP I protein levels at 37°C |
| IS0016 | Lower | Lower | Lower | A272E in RNA Pol alpha factor | Mutation may impact RNA Pol binding to some promoters [1] |

^1^ Relative pYV PCN was determined using a luciferase PCN assay with luminescence
 normalized to OD_600_.

^2^ Changes in pYV PCN and T3SS activity relative to the parental strain. The parental strain
 for *pil::pNQ* is wildtype *Y. pseudotuberculosis* YPIII/pIBX and the parental strain for all
 suppressor isolates shown is *pil::pNQ* YPIII/pIBX.

^3^ T3SS activity was determined via a secretion assay of cells grown at 37°C in low calcium
 media.

**Table S2. *Y. pseudotuberculosis* strains used in this study**

| Strain | Background | Mutation(s) | Ref |
| --- | --- | --- | --- |
| Wildtype (WT) | IP2666pIB1 | Naturally lacks full-length YopT | [2] |
| *pil::Tn* | IP2666pIB1 | Tn5 insertion in *pil,* wildtype background | This work |
| *∆pcnB* | IP2666pIB1 | *∆pcnB* | This work |
| *pil::Tn^∆pcnB^* | IP2666pIB1 | *∆pcnB, pil::Tn* background | This work |
| PAP I^His^ | IP2666pIB1 | PAP I with C-6xHis tag | This work |
| PAP I^D2A^ | IP2666pIB1 | D114A+D116A mutations in PAP I (C-6xHis) | This work |
| PAP I^L291R^ | IP2666pIB1 | L291R mutation in PAP I (C-6xHis) | This work |
| PAP I^L291A^ | IP2666pIB1 | L291A mutation in PAP I (C-6xHis) | This work |
| PAP I^FLAG^ | IP2666pIB1 | PAP I with C-3xFLAG tag | This work |
| PAP I^D2A-FLAG^ | IP2666pIB1 | D114A+D116A mutations in PAPI(C-3xFLAG) | This work |
| PAP I^L291R-FLAG^ | IP2666pIB1 | L291R mutation in PAP I (C-3xFLAG) | This work |
| PAP I^L291A-FLAG^ | IP2666pIB1 | L291A mutation in PAP I (C-3xFLAG) | This work |
| *ΔyopHEMOJ* | IP2666pIB1 | *ΔyopHEMOJ* | [3] |
| *ΔyscNU* | IP2666pIB1 | *ΔyscNU* | [4] |
| pYV- | IP2666 | Cured of pYV | [3] |
| *pil::Tn* | IP2666pIB1 | Tn5 insertion in *pil, ∆yopEMOJ* background | This work |
| Wildtype (WT) | YPIII pIBX | Naturally lacks full-length YopT; pIBX is pYV encoding two copies of the *luxCDABE* operon and a kanamycin resistance gene | [5] |
| *pil::pNQ* | YPIII pIBX | pNQ insertion in *pil* | This work |
| *∆pcnB* | YPIII pIBX | *∆pcnB* | This work |
| ParB-GFP | YPIII pIBX | ParB-C-msfGFP | This work |
| *∆pcnB*^ParB-GFP^ | YPIII pIBX | *∆pcnB* ParB-C-msfGFP | This work |
| PAP I^His^ | YPIII pIBX | PAP I with C-6xHis tag | This work |
| PAP I^D2A^ | YPIII pIBX | D114A+D116A mutations in PAP I (C-6xHis) | This work |
| PAP I^L291R^ | YPIII pIBX | L291R mutation in PAP I (C-6xHis) | This work |
| PAP I^L291A^ | YPIII pIBX | L291A mutation in PAP I (C-6xHis) | This work |
| ∆*ipaH2.5::tet^R^* | M90T | ∆*ipaH2.5* | [6] |
| ∆*ipaH2.5*∆*pcnB* | M90T | ∆*ipaH2.5*∆*pcnB* | This work |
| VP- | M90T | BS176 | [7] |

**Table S3. Primers used in this study**

| **Name** | **Primer Sequence** | **Ref** |
| --- | --- | --- |
| **Primers used to make pCVD442::*∆pcnB*** | |  |
| FpcnB_500up_pCVD | caacataaaggtgaatcccatatgAGAAGATTTTATTATCCGTCG | This work |
| RpcnB_500up_pCVD | ggtaaaattaAATGGTACACCTCGATAG | This work |
| FpcnB_500d_pCVD | gtgtaccattTAATTTTACCATGATCCGGGTC | This work |
| RpcnB_500d_pCVD | acctggcacggctgggacggaagtcTGAGCCGGCGATATTACC | This work |
| **Primers used to make pCVD442::PAPI^His^** | |  |
| FpcnB_pET28 | agtggtggtggtggtggtgctcgagTACCCCTTCTTTACGGGG | This work |
| RpcnB_pET28 | actttaagaaggagatataccatggATTTTTACCCGAGTAGCC | This work |
| FpcnB_500up_His | gaggtgtaccATTTTTACCCGAGTAGCCAATTTC | This work |
| RpcnB_500up_His | catggtaaaaTCAGTGGTGGTGGTGGTG | This work |
| FpET_pB_His_300d | caacataaaggtgaatcccatatgAGGGGAATAAGCTCTCCAAG | This work |
| RpET_pB_His_300d | gggtaaaaatGGTACACCTCGATAGTGG | This work |
| FpcnB_300d_pCVD | ccaccactgaTTTTACCATGATCCGGGTC | This work |
| RpcnB_300d_pCVD | tgacagtctccggaagacggTGAGCCGGCGATATTACC | This work |
| **Primers used to make pCVD442::PAPI^FLAG^** | |  |
| FpcnB_FLAG | caacataaaggtgaatcccaTGAAAAATACCGACCACC | This work |
| RpcnB_FLAG | ctttgtagtcTACCCCTTCTTTACGGGG | This work |
| FpET_FLAG_pcnB | agaaggggtaGACTACAAAGACCATGACG | This work |
| RpET_FLAG_pcnB | ggtaaaattaCATATGGTACCAGCTGCAG | This work |
| FpcnB_500d_pCVD | gtaccatatgTAATTTTACCATGATCCGGGTC | This work |
| RpcnB_500d_pCVD | tgacagtctccggaagacggTGAGCCGGCGATATTACC | This work |
| **Primers used to make pCVD442::ParB-msfGFP** | |  |
| FparB_GFP | caacataaaggtgaatcccatatgTTGCTAATGAGTATCGTC | This work |
| RparB_GFP | cgccttttgaCAAAGAATGTTCCTTTGC | This work |
| FpKD_GFP_parB | acattctttgTCAAAAGGCGAAGAACTTTTTAC | This work |
| RpKD_GFP_parB | tattcaggcaTTATTTATACAATTCATCCATTCCATGAGTGAT TCCTGCCGCAGTGACAAATTC | This work |
| FparB_500d_pCVD | gtataaataaTGCCTGAATAAGATCAGAAC | This work |
| RparB_500d_pCVD | tgacagtctccggaagacggAATCTCCCTAAAGCTATCAC | This work |
| **Q5 mutagenesis primers used to introduce L291R mutation** | |  |
| FpB_L291R_pCVD | ACTCAAGCTGaggCAATCCGGCTAC | This work |
| RpB_L291R_pCVD | GACTCCTCAAACAGGCGG | This work |
| **Q5 mutagenesis primers used to introduce L291A mutation** | |  |
| FpB_L291A_pCVD | ACTCAAGCTGgcgCAATCCGGCTAC | This work |
| RpB_L291A_pCVD | GACTCCTCAAACAGGCGG | This work |
| **Q5 mutagenesis primers used to introduce D114A + D116A (D2A) mutations** | | |
| FpB_D2A_pCVD | tcgctATCACCACCAGCGC | This work |
| RpB_D2A_pCVD | aagcTTTGGGTTTTCTGCCC | This work |
| **Primers used to make pTrc99::PAP I** | | |
| FpcnB_pTrc | atttcacacaggaaacagaccatggATTTTTACCCGAGTAGCCAATTTC | This work |
| RpcnB_pTrc | tgcatgcctgcaggtcgactctagaCAGCGCGATATAGACCCG | This work |
| **Primers used for ddPCR** | | |
| F_Chrom_ddPCR | CCTCACCGATACCGAACGAG | [8] |
| R_Chrom_ddPCR | GTCAGCAGGATAGGGCTACC | [8] |
| F_pYV_ddPCR | CTCTTTGACCTCGGCTTGAG | [8] |
| R_pYV_ddPCR | CGCAGCCGTTAGGACAAATG | [8] |
| **Primers used to make ∆*ipaH2.5*∆*pcnB Shigella*** | | |
| pcnb_F | ggcagaagcacactggcagg | This work |
| pcnb_R | gtggtccccagcgttcagc | This work |
| pcnB_R_op | gacttcacgcaacgtctcccc | This work |
| K2_wanner | cggtgccctgaatgaactgc | [9] |

**Table S4. Plasmids used in this study**

| **Vector/Plasmid** | **Insert** | **Ref** |
| --- | --- | --- |
| pCVD442::empty | None [SacB^+^] | [10] |
| pET28::empty | -C-3xFLAG and -C-6xHis | [11] |
| pKD13-msfGFP | -C-msfGFP | ^[12]^ |
| pCVD442:: *∆pcnB* | *∆pcnB* | This work |
| pCVD442::PAP I^His^ | PAP I-C-6xHis | This work |
| pCVD442::PAP I^L291R^ | PAP I^L291R-C-6xHis^ | This work |
| pCVD442::PAP I^L291A^ | PAP I^L291A-C-6xHis^ | This work |
| pCVD442::PAP I^D2A^ | PAP I^D2A-C-6xHis^ | This work |
| pCVD442::PAP I^FLAG^ | PAP I-C-3xFLAG | This work |
| pCVD442::PAP I^L291R-FLAG^ | PAP I^L291R-C-3xFLAG^ | This work |
| pCVD442::PAP I^L291A-FLAG^ | PAP I^L291A-C-3xFLAG^ | This work |
| pCVD442::PAP I^D2A-FLAG^ | PAP I^D2A-C-3xFLAG^ | This work |
| pCVD442::ParB-msfGFP | ParB-C-msfGFP | This work |
| pTrc99A::empty | None [IPTG-inducible T7] | [13] |
| pTrc99::PAP I | PAP I | This work |
| pGFP-uv | GFPuv | [14] |
| pNF06::ccdAB | ccdAB | [15] |

1. Belin D, Costafrolaz J, Silva F. AraC Functional Suppressors of Mutations in the C-Terminal Domain of the RpoA Subunit of the *Escherichia coli* RNA Polymerase. Microorganisms. 2024;12(9). Epub 20240923. doi: 10.3390/microorganisms12091928. PubMed PMID: 39338602; PubMed Central PMCID: PMCPMC11434276.

2. Bliska JB, Guan KL, Dixon JE, Falkow S. Tyrosine phosphate hydrolysis of host proteins by an essential *Yersinia* virulence determinant. Proc Natl Acad Sci U S A. 1991;88(4):1187-91. doi: 10.1073/pnas.88.4.1187. PubMed PMID: 1705028; PubMed Central PMCID: PMCPMC50982.

3. Auerbuch V, Golenbock DT, Isberg RR. Innate immune recognition of *Yersinia pseudotuberculosis* type III secretion. PLoS Pathog. 2009;5(12):e1000686. Epub 20091204. doi: 10.1371/journal.ppat.1000686. PubMed PMID: 19997504; PubMed Central PMCID: PMCPMC2779593.

4. Balada-Llasat JM, Mecsas J. *Yersinia* has a tropism for B and T cell zones of lymph nodes that is independent of the type III secretion system. PLoS Pathog. 2006;2(9):e86. doi: 10.1371/journal.ppat.0020086. PubMed PMID: 16948531; PubMed Central PMCID: PMCPMC1557584.

5. Fahlgren A, Avican K, Westermark L, Nordfelth R, Fallman M. Colonization of cecum is important for development of persistent infection by *Yersinia pseudotuberculosis*. Infect Immun. 2014;82(8):3471-82. Epub 20140602. doi: 10.1128/IAI.01793-14. PubMed PMID: 24891107; PubMed Central PMCID: PMCPMC4136198.

6. Sidik S, Kottwitz H, Benjamin J, Ryu J, Jarrar A, Garduno R, et al. A *Shigella flexneri* virulence plasmid encoded factor controls production of outer membrane vesicles. G3 (Bethesda). 2014;4(12):2493-503. Epub 20141105. doi: 10.1534/g3.114.014381. PubMed PMID: 25378474; PubMed Central PMCID: PMCPMC4267944.

7. Sansonetti PJ, Mounier J. Metabolic events mediating early killing of host cells infected by *Shigella flexneri*. Microb Pathog. 1987;3(1):53-61. doi: 10.1016/0882-4010(87)90037-4. PubMed PMID: 2848171.

8. Schneiders S, Hechard T, Edgren T, Avican K, Fallman M, Fahlgren A, et al. Spatiotemporal Variations in Growth Rate and Virulence Plasmid Copy Number during *Yersinia pseudotuberculosis* Infection. Infect Immun. 2021;89(4). Epub 20210317. doi: 10.1128/IAI.00710-20. PubMed PMID: 33495272; PubMed Central PMCID: PMCPMC8090943.

9. Datsenko KA, Wanner BL. One-step inactivation of chromosomal genes in *Escherichia coli* K-12 using PCR products. Proc Natl Acad Sci U S A. 2000;97(12):6640-5. doi: 10.1073/pnas.120163297. PubMed PMID: 10829079; PubMed Central PMCID: PMCPMC18686.

10. Donnenberg MS, Kaper JB. Construction of an eae deletion mutant of enteropathogenic *Escherichia coli* by using a positive-selection suicide vector. Infect Immun. 1991;59(12):4310-7. doi: 10.1128/iai.59.12.4310-4317.1991. PubMed PMID: 1937792; PubMed Central PMCID: PMCPMC259042.

11. Shis DL, Bennett MR. Library of synthetic transcriptional AND gates built with split T7 RNA polymerase mutants. Proc Natl Acad Sci U S A. 2013;110(13):5028-33. Epub 20130311. doi: 10.1073/pnas.1220157110. PubMed PMID: 23479654; PubMed Central PMCID: PMCPMC3612686.

12. Gray WT, Govers SK, Xiang Y, Parry BR, Campos M, Kim S, et al. Nucleoid Size Scaling and Intracellular Organization of Translation across Bacteria. Cell. 2019;177(6):1632-48 e20. doi: 10.1016/j.cell.2019.05.017. PubMed PMID: 31150626; PubMed Central PMCID: PMCPMC6629263.

13. Amann E, Ochs B, Abel KJ. Tightly regulated tac promoter vectors useful for the expression of unfused and fused proteins in *Escherichia coli*. Gene. 1988;69(2):301-15. doi: 10.1016/0378-1119(88)90440-4. PubMed PMID: 3069586.

14. Million-Weaver S, Alexander DL, Allen JM, Camps M. Quantifying plasmid copy number to investigate plasmid dosage effects associated with directed protein evolution. Methods Mol Biol. 2012;834:33-48. doi: 10.1007/978-1-61779-483-4_3. PubMed PMID: 22144351; PubMed Central PMCID: PMCPMC3804865.

15. Jurenas D, Fraikin N, Goormaghtigh F, De Bruyn P, Vandervelde A, Zedek S, et al. Bistable Expression of a Toxin-Antitoxin System Located in a Cryptic Prophage of *Escherichia coli* O157:H7. mBio. 2021;12(6):e0294721. Epub 20211130. doi: 10.1128/mBio.02947-21. PubMed PMID: 34844426; PubMed Central PMCID: PMCPMC8630535.
