# Supplementary material for: The polyadenylase PAPI is required for virulence plasmid maintenance in pathogenic bacteria
Source: bioRxiv. 2024 Oct 11:2024.10.11.617751. Preprint. [Version 1] doi: 10.1101/2024.10.11.617751 (PMC11482874; doi:10.1101/2024.10.11.617751)
Supplement: Supplementary file 1 [file NIHPP2024.10.11.617751v1-supplement-1.pdf]

bioRxiv preprint doi: https://doi.org/10.1101/2024.10.11.617751; this version posted October 11, 2024. The copyright holder for this preprint (which was not certified by peer review) is the author/funder, who has granted bioRxiv a license to display the preprint in perpetuity. It is made available under aCC-BY 4.0 International license.

Supporting Information

Figure S1. A transposon insertion into *pil* leads to a constitutively high pYV PCN, elevated T3SS expression, and a severe growth defect under T3SS-inducing conditions. **(A)** HEK293T cells expressing an NFκB luciferase reporter were left uninfected (uninf) or were infected with an effectorless Δyop6 strain (Δ*yopHEMOJ*), a Δ*yscNU* T3SS-deficient strain, or *pil::Tn* in the *Y. pseudotuberculosis* IP2666pIB1 background. Averages of three independent replicates ± standard error the mean are shown (Student t-test, p<0.04). **(B)** Schematic representation of the location of *pil*. The *pil* locus is located ~123bp away from the *copB* gene. **(C)** Wildtype, *pil::Tn*, and Δ*pil* IP2666pIB1 were grown at 37°C/low calcium and secreted T3SS cargo proteins (Yops) were precipitated and visualized by Coomassie blue staining. Bovine serum albumin (BSA) serves as a loading and precipitation control. Data shown is representative of three independent replicates. **(D-E)** Relative pYV PCN was estimated for strains in the YPIII/pIBX background of *Y. pseudotuberculosis* using a luciferase plasmid copy number assay. For each timepoint, luminescence was measured and normalized to cell density

bioRxiv preprint doi: https://doi.org/10.1101/2024.10.11.617751; this version posted October 11, 2024. The copyright holder for this preprint (which was not certified by peer review) is the author/funder, who has granted bioRxiv a license to display the preprint in perpetuity. It is made available under aCC-BY 4.0 International license.

(OD_{600}). Representative data from three independent experiments are shown. **(E)** Serial dilutions for wildtype YPIII/pIBX and the *pil*::pNQ congenic *Y. pseudotuberculosis* strains were spotted onto low calcium agar plates for ~16 hours at 37°C before imaging. The yellow arrow indicates an example of a large colony in the *pil*::pNQ mutant background that represents a candidate suppressor mutant.

Figure S2. T3SS gene mRNA levels are higher in the *pil*::Tn mutant and lower in the Δ*pcnB* mutant compared to wildtype. Heat maps displaying relative expression of pYV-encoded genes from RNA-seq analysis of *Y. pseudotuberculosis* IP2666pIB1 strains grown at 26°C or 37°C/low calcium.

Figure S3. The *pil*::Tn strain exhibits normal temperature and calcium regulation of T3SS activity. Strains were grown in regular LB or low calcium LB media, at either 26°C or at 37°C. Secreted proteins in the supernatant were precipitated and visualized by SDS-PAGE and Coomassie blue staining. Bovine serum albumin (BSA) was added as a loading and precipitation control. Data shown is representative data of three biological replicates.

Figure S4. Transposon insertion in the *pil* locus alters RNA-Seq read patterns at the *cop-rep* locus. Reads from RNA-seq analysis mapped to the *cop-rep* locus and adjacent *pil* locus from wildtype and *pil*::Tn (*pil*) *Y. pseudotuberculosis* IP2666pIB1 grown at 26°C or 37°C/low calcium. The putative *pil* locus is indicated by a black box and the approximate location of the Tn insertion is indicated with an arrow.

bioRxiv preprint doi: https://doi.org/10.1101/2024.10.11.617751; this version posted October 11, 2024. The copyright holder for this preprint (which was not certified by peer review) is the author/funder, who has granted bioRxiv a license to display the preprint in perpetuity. It is made available under aCC-BY 4.0 International license.

Figure S5. The *pcnB* gene is required for plasmid-mediated antibiotic resistance in *Y. pseudotuberculosis.* Wildtype and Δ*pcnB* *Y. pseudotuberculosis* IP2666pIB1 harboring either pBAD33 or pACYC184 were grown overnight in LB supplemented with 20 µg/mL chloramphenicol, and the overnight cultures spotted onto LB agar lacking or containing chloramphenicol to assess plasmid retention. Plates were incubated at 26°C for ~16 hours before imaging. Each experiment was carried out for a total of two independent experiments.

Figure S6. *Y. pseudotuberculosis* isolated from infected livers demonstrate similar plasmid loss and T3SS activity phenotypes as their parental strains. Independent colony forming units from infected mouse livers were isolated and assessed for their ability to retain pYV and carry out T3SS activity. WT-3 and WT-5 are wildtype IP2666pIB1-derived isolates from two different mice. Δ*pcnB*-2-9, Δ*pcnB*-2-10, Δ*pcnB*-3-1, and Δ*pcnB*-3-2 are Δ*pcnB*-derived isolates from two different mice. **(A)** Liver isolates and their parental strains were incubated overnight at 26˚C in LB or 37˚C in LB/low calcium media. Colonies were patched onto low calcium media containing Congo red to induce T3SS activity and assess the percentage of colonies that retained pYV (i.e.-red colonies). **(B)** Liver isolates and their parental strains were incubated in LB low calcium media at 37˚C, and secreted proteins visualized by Coomassie blue staining. Bovine serum albumin (BSA) was added as a loading and precipitation control. Data shown is representative of two independent experiments.

Figure S7. Loss of PAP I does not affect *Y. pseudotuberculosis* growth following stress exposure. (A) Wildtype and Δ*pcnB* *Y. pseudotuberculosis* IP2666pIB1 cultures were grown to mid-log phase at 26°C before shifting to 42°C for 1 hour to induce heat shock. Spot dilutions were plated onto LB plates and incubated at 26°C for ~16 hours before imaging. **(B)** Strains in

bioRxiv preprint doi: https://doi.org/10.1101/2024.10.11.617751; this version posted October 11, 2024. The copyright holder for this preprint (which was not certified by peer review) is the author/funder, who has granted bioRxiv a license to display the preprint in perpetuity. It is made available under aCC-BY 4.0 International license.

the IP2666pIB1 background were grown at 26°C overnight (stationary phase) before diluting and spotting into LB plates. Plates were incubated at 26°C for 4 hours before transferring to 4°C and allowing growth for ~2 days prior to imaging. **(C)** Wildtype and Δ*pcnB Y. pseudotuberculosis* IP2666pIB1 were grown at 37°C (low calcium) to mid-log phase before inducing stress. Growth was assayed by plating spot dilutions following stress exposure. Stress was induced by adjusting media to pH=3.0 to induce acid shock or by addition of 800Mm $NaCl_2$ to induce osmotic shock.

Figure S8. PAP I from *Y. pseudotuberculosis* and *Y. pestis* are 100% conserved. Predicted PAP I sequences from *Y. pseudotuberculosis* IP2666pIB1 and *Y. pestis* KIM5.

Figure S9. *Y. pseudotuberculosis* displays normal T3SS activity in EZ-RDM low calcium media. *Y. pseudotuberculosis* IP2666pIB1 wildtype, Δ*pcnB*, and pYV- strains were grown in either low calcium LB or low calcium EZ-RDM media. The pYV- strain cured of pYV was used as a negative control [103]. Secreted proteins were visualized by Coomassie blue staining. Bovine serum albumin (BSA) was added as a loading and precipitation control. Data shown is representative of two independent experiments.

Table S1. Phenotypic categories and mutations identified in suppressor screen.

Table S2. *Y. pseudotuberculosis* strains used in this study.

Table S3. Primers used in this study.

Table S4. Plasmids used in this study.

bioRxiv preprint doi: https://doi.org/10.1101/2024.10.11.617751; this version posted October 11, 2024. The copyright holder for this preprint (which was not certified by peer review) is the author/funder, who has granted bioRxiv a license to display the preprint in perpetuity. It is made available under aCC-BY 4.0 International license.

Dataset S1. Transcriptome analysis of *Y. pseudotuberculosis* IP2666pIB1, *pil*::Tn, Δ*pcnB*, and *pil*::Tn/Δ*pcnB* congenic strains.
